# An N-terminal helix of Lsm11 stabilizes CPSF73 in U7 snRNP for histone pre-mRNA 3′-end processing

**DOI:** 10.1093/nar/gkaf1442

**Published:** 2026-01-06

**Authors:** Anthony Desotell, William F Marzluff, Zbigniew Dominski, Liang Tong

**Affiliations:** Department of Biological Sciences, Columbia University, New York, NY 10027, United States; Integrative Program for Biological and Genome Sciences, University of North Carolina at Chapel Hill, Chapel Hill, NC 27599, United States; Department of Biochemistry and Biophysics, University of North Carolina at Chapel Hill, Chapel Hill, NC 27599, United States; Integrative Program for Biological and Genome Sciences, University of North Carolina at Chapel Hill, Chapel Hill, NC 27599, United States; Department of Biochemistry and Biophysics, University of North Carolina at Chapel Hill, Chapel Hill, NC 27599, United States; Department of Biological Sciences, Columbia University, New York, NY 10027, United States

## Abstract

The U7 snRNP (small nuclear ribonucleoprotein) is responsible for the 3′-end processing of replication-dependent histone messenger RNA precursors (pre-mRNAs). A helix in the Lsm11 N-terminal extension contacts the metallo-β-lactamase domain of the U7 snRNP endonuclease CPSF73. We mutated or deleted this helix and found that the mutant machineries had substantially reduced cleavage activity toward the pre-mRNA. Our cryo-electron microscopy (cryo-EM) studies indicated that the helix was important for helping to hold CPSF73 in its correct position for the cleavage reaction. We also reconstituted a wild-type U7 snRNP in complex with a methylated, noncleavable pre-mRNA. We observed that CPSF73 could achieve an open conformation independent of RNA binding to its active site. Finally, we found that a previously uninterpreted EM density for a small helix at the CPSF73-CPSF100 interface belonged to the C-terminal end of CstF77, copurified from insect cells and highly conserved among CstF77 homologs. This CstF77 binding site had a small effect on the cleavage activity of U7 snRNP. Overall, our studies have revealed the importance of the conserved helix in the Lsm11 N-terminal extension for U7 snRNP, provided structural evidence that CPSF73 can achieve an open, active conformation without RNA binding in its active site, and identified a previously unknown binding site for CstF77 in CPSF100.

## Graphical Abstract

**Graphical Abstract:**
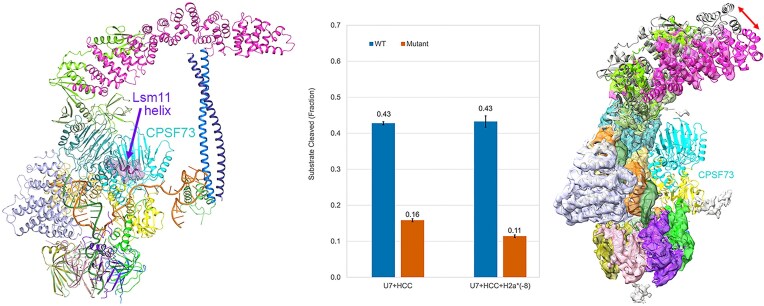

## Introduction

Eukaryotic messenger RNA precursors (pre-mRNAs) undergo extensive co-transcriptional processing in the nucleus before they become mature mRNAs and are exported to the cytoplasm for translation into proteins. Most pre-mRNAs are cleaved and then polyadenylated at their 3′ end. This canonical 3′-end processing is catalyzed by a large machinery of >15 different protein factors [1–3]. In contrast, metazoan replication-dependent histone pre-mRNAs are cleaved at their 3′ end but not polyadenylated, and a distinct machinery, the U7 snRNP (small nuclear ribonucleoprotein), catalyzes this processing [4–6].

**Figure 1.**
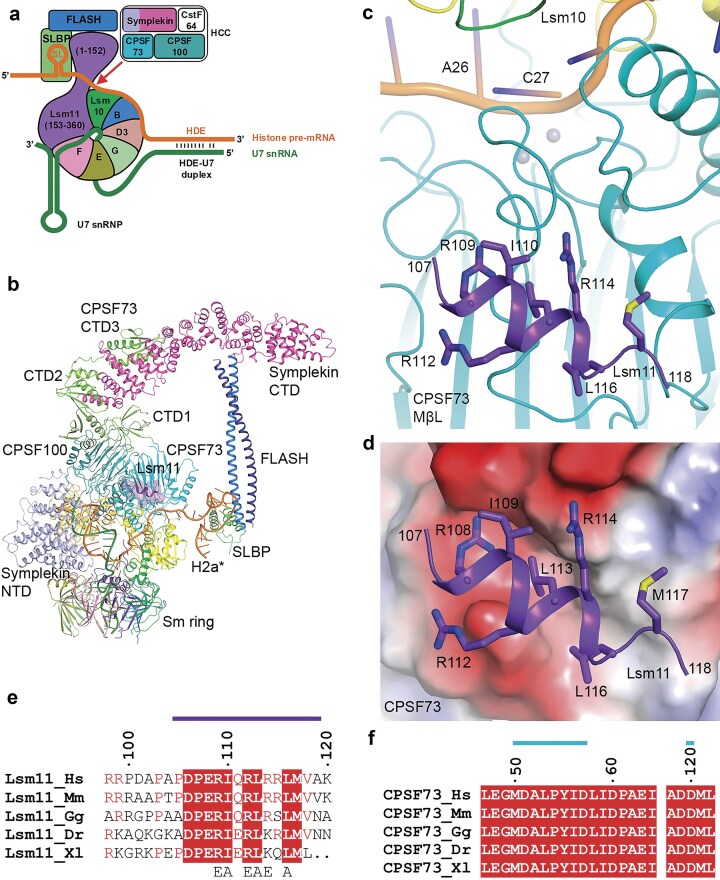
A segment in the N-terminal extension of Lsm11 contacts CPSF73 in the U7 snRNP. (**a**) Schematic drawing of U7 snRNP and its recognition of histone pre-mRNA. The subunits are given different colors and labeled. (**b**) Overall structure of the U7 snRNP, colored as in panel (a). The core of the structure is determined based on EM density, and the symplekin CTD and CPSF73 CTD3 structures are based on docking AlphaFold models into the lower resolution EM density at the periphery. The Lsm11 N-terminal segment is highlighted with a purple transparent surface. The H2a* pre-mRNA substrate is in orange, and the U7 snRNA in dark green. (**c**) Residues 107–118 in the N-terminal extension of Lsm11 (purple) are in contact with the metallo-β-lactamase (MβL) domain of CPSF73 (cyan). The pre-mRNA in the active site of CPSF73 is shown in orange. The two metal ions are shown as spheres in gray. (**d**) Electrostatic surface of CPSF73 (red: negatively charged; blue: positively charged) near the binding site for the Lsm11 segment. (**e**) Sequence conservation of residues in the Lsm11 segment in contact with CPSF73. Strictly conserved residues are highlighted with a red background. Residues in the binding site are indicated with the purple bar above. Residues selected for mutagenesis are indicated below. Hs: human; Mm: mouse; Gg: chicken; Dr: Zebrafish; Xl: frog. (**f**) Sequence conservation of CPSF73 residues that are in contact with the Lsm11 segment (cyan bar).

The active U7 snRNP contains the U7 snRNA and seven Sm proteins that together recognize this RNA (Fig. 1a). Among them, Lsm10 and Lsm11 are unique to U7 snRNP, and Lsm11 has a long N-terminal extension that is required for interacting with the protein FLASH [7–10]. Together, Lsm11 and FLASH recruit the histone pre-mRNA cleavage complex (HCC), comprised of CPSF73, CPSF100, symplekin, and CstF64 [8, 9, 11]. HCC is equivalent to the mammalian cleavage factor (mCF; with CPSF73, CPSF100, and symplekin) in the canonical machinery [12, 13], and CPSF73 is the endonuclease that catalyzes the cleavage reaction in both machineries [14, 15]. Histone pre-mRNAs contain a conserved stem-loop (SL) upstream of the cleavage site that is recognized by SL binding protein (SLBP), and a purine-rich histone downstream element (HDE) that base pairs with the 5′ end of the U7 snRNA, forming an HDE–U7 duplex (Fig. 1a).

We recently reported the cryo-electron microscopy (cryo-EM) structure of a fully reconstituted, active U7 snRNP in complex with a model histone pre-mRNA substrate (H2a*) (Fig. 1b) [16]. The HDE–U7 duplex is bound by symplekin N-terminal domain (NTD), CPSF73 and CPSF100. CPSF73 is in an open state, with the pre-mRNA substrate captured in the active site and poised for the cleavage reaction. In comparison, the symplekin C-terminal domain (CTD), FLASH, SLBP, and SL are located at the periphery of the machinery and are highly flexible, and only a low-resolution EM map could be obtained for them. Despite the reduced resolution, the AlphaFold2 model [17] for symplekin CTD can be readily fitted into the EM density, which also shows interactions with the CTD3 of CPSF73. A model for the entire U7 snRNP in complex with a histone pre-mRNA substrate can thus be produced based on the EM density and aided by AlphaFold2 predictions where necessary (Fig. 1b). The function of CstF64 in the HCC is not known. It is not observed in the EM density of the U7 snRNP, suggesting that it is flexible in the machinery.

The EM density also reveals that residues 107–118 in the N-terminal extension of Lsm11 contact the MβL domain of CPSF73 (Fig. 1b and c). The binding site is ~25 Å from the active site of CPSF73, and therefore this Lsm11 segment is unlikely to directly affect catalysis. This segment has primarily the structure of an α helix, and many of its side chains have intimate contacts with CPSF73 (Fig. 1c and d). The segment is located in an electronegative surface depression of CPSF73, which offers favorable interactions with three arginine residues in Lsm11 (Fig. 1d). Residues in this segment are highly conserved among vertebrate Lsm11 orthologs (Fig. 1e), as are the CPSF73 residues in this interface (Fig. 1f). However, it is not known whether this helix and its interaction with CPSF73 have any effect on the activity of the U7 snRNP. This binding site overlaps with that between the yeast CPSF73 ortholog (Ysh1) and the yeast RBBP6 homolog (Mpe1) [16, 18]. Mpe1 is an important component of the yeast 3′-end processing machinery and RBBP6 is required for the cleavage activity of the mammalian canonical machinery [19, 20], and we hypothesized that this Lsm11 segment might also contribute to the cleavage activity of the U7 snRNP.

To assess the functional importance of this Lsm11 segment in the cleavage activity of U7 snRNP, we have mutated six of its residues or deleted this segment to disrupt the interaction with CPSF73 (Fig. 1e). Our biochemical studies show that the U7 snRNP incorporating such a mutant Lsm11 has substantially weaker cleavage activity compared to the wild-type machinery. Our cryo-EM studies show that the CPSF73 catalytic segment is not observed in a large collection of the particles, suggesting that its position is flexible in the mutant. These observations indicate that the Lsm11 segment is important for helping to hold CPSF73 in its correct position in the U7 snRNP.

The pre-mRNA substrate was captured in the active site of CPSF73 in our earlier structure of the reconstituted U7 snRNP [16] as well as the structure containing the Lsm11 mutant reported here. However, only a small percentage of the particles were in this state based on the cryo-EM data analysis. We hypothesized that we might be able to increase the percentage of particles in this active state by using a noncleavable pre-mRNA substrate. Such a substrate might also stabilize the overall machinery, which might allow us to obtain a better-quality EM map for the entire U7 snRNP. Therefore, we reconstituted a U7 snRNP with a noncleavable pre-mRNA substrate, where the 2′ hydroxyl of the ribose of five nucleotides spanning the cleavage site was methylated [8, 11, 21]. We found that CPSF73 was in an open state, but the pre-mRNA was not located in the active site.

In the U7 snRNP structure that we reported earlier [16], there was a small piece of EM density at the interface between CPSF73 and CPSF100 that could not be explained. This density is also observed in the new EM maps reported here. Aided by AlphaFold-Multimer [22, 23], we have found that this density corresponds to residues 715–722 at the C-terminus of CstF77. Specifically, this is *Trichoplusia ni* CstF77 that copurified with the machinery from insect cells, as we did not express human CstF77 in these experiments. CstF77 is a subunit of the cleavage stimulation factor (CstF) that recognizes the downstream element of the pre-mRNA in the canonical machinery. CstF77 may be a scaffold that facilitates the dimerization of this factor [24, 25]. Another subunit of CstF, CstF64, is a well-established component of the U7 snRNP, as a subunit of the HCC although its function in the HCC is not known. The C-terminal residues of CstF77 that contact CPSF73–CPSF100 are highly conserved among its homologs. Our mutagenesis studies show that this binding site has only a small effect on the U7 snRNP cleavage activity, consistent with earlier data on the U7 snRNP that did not show CstF77 as a component [11, 26].

## Materials and methods

### Protein expression and purification

Human Lsm11 carrying six mutations R109E/I110A/R112E/L113A/R114E/L116A in the N-terminal segment or with residues 102–120 deleted was generated with an oligo cassette containing the necessary nucleotide changes, followed by Gibson assembly [27] into polymerase chain reaction-linearized vector. All proteins of the U7 snRNP were expressed and purified following protocols described earlier [16, 28].

Residues 666–717 of human CstF77 were cloned into the pET28a vector (Novagen), which also introduced either a His-SUMO or a His-MBP (maltose binding protein) tag at the N terminus. Two overlapping DNA duplex oligos encoding residues 666–717 were obtained (Azenta) (Supplementary Table S1). They were mixed together and treated with T4 polynucleotide kinase for ligation into the vector with T4 ligase. The insert was verified by sequencing, and the plasmid used to transform BL21(DE3) Star cells (Novagen). The cells were grown to OD_600_ of 0.8 and induced with 0.4 mM IPTG (isopropyl β-D-1-thiogalactopyranoside) at 16°C for 18 h. Recombinant protein was purified by nickel–agarose affinity (Qiagen) followed by gel filtration (Superdex 200, Cytiva). Relevant fractions were collected, concentrated, flash frozen, and stored at −80°C.

Residues 673–727 of *T. ni* CstF77 were expressed and purified as an MBP fusion protein following the same protocols.

For co-expression with HCC in insect cells, residues 666–717 of human CstF77 or residues 673–727 of *T. ni* CstF77 were cloned into the pFastBac 438A vector with Gibson assembly. The proteins carried an MBP tag but no His tag.

### RNAs

The U7 snRNA was synthesized by *in vitro* transcription following protocols described earlier [26]. A 60-mer 5′ fluorescein (FAM)-labeled mouse histone 2 pre-mRNA model substrate (H2a*) was obtained from Integrated DNA Technologies (IDT). The RNA sequences are given in Supplementary Table S1. A 52-mer substrate lacking the eight 3′ nucleotides of H2a* [H2a*(-8)] was also obtained from IDT, either with 5′ FAM label or doubly labeled with TAMRA at 5′ end and FAM at 3′ end. A 56-mer substrate methylated on the 2′ hydroxyl group of five nucleotides around the cleavage site and lacking four 3′ nucleotides of H2a* [H2a*m(-4)] was obtained from Dharmacon.

### *In vitro* assembly of U7 Sm core for biochemical studies

The U7 Sm core complex was reconstituted using wild-type, sextuple mutant or deletion mutant Lsm11 as described earlier [16, 28]. SLBP was not included in the assembly of the U7 Sm core for biochemical studies so that we could test the activity of the machinery with and without it. The U7 Sm core complex was purified by gel filtration using Superose 6 Increase 10/300 GL column (Cytiva). Fractions of interest were concentrated, flash froze in liquid nitrogen, and stored at −80°C.

### *In vitro* processing assays

Cleavage reactions containing 1 μM U7 Sm core containing wild-type, sextuple mutant or deletion mutant Lsm11 and 1 μM HCC, with or without 1 μM SLBP were carried out in a 10-μl volume containing 15 mM HEPES (pH 8.0), 75 mM KCl, 15% (v/v) glycerol, 20 mM ethylenediaminetetraacetic acid (EDTA; pH 8.0), and 1 U RNasin Plus ribonuclease inhibitor (Promega). Some of the reactions also contained 0.1 μM H2a*(-8). Reactions were incubated at 30°C for 1 h and then stopped by addition of 10 μl 2× RNA loading dye (NEB). Samples were then boiled at 95°C for 15 min and cooled to room temperature. The mixture was separated on 15% urea gels and visualized using the 488 and 546-fluorescence channel of a ChemiDoc (Bio-Rad).

Cleavage products were quantified using Fiji [29]. Raw images were converted to 8-bit and band intensities were calculated for all major bands in each lane. The fraction cleaved was calculated by dividing the intensity of the cleavage product (26-mer) by the sum of the cleavage product and the uncleaved H2a* and H2a*(-8) (when present) within each lane. Each condition was tested in triplicates.

### *In vitro* reconstitution of the U7 snRNP for EM studies

U7 Sm ring was reconstituted using wild-type or mutant Lsm11 following protocols described earlier [28]. Separately, a U7 Sm ring with wild-type Lsm11 and the methylated noncleavable H2a*m(-4) pre-mRNA was assembled. The U7 Sm ring used in all structural studies also included SLBP. The Sm ring was purified by gel filtration using Superose 6 Increase 10/300 GL (Cytiva). Fractions of interest were concentrated and used immediately to assemble the full U7 machinery.

Purified HCC was mixed with wild-type U7 Sm ring with methylated H2a*m(-4) RNA and excess FLASH in a 1:1.2:2.4 molar ratio and incubated on ice for 60 min. Sample was then injected into Superose 6 Increase 3.2/300 column (Cytiva), equilibrated with a buffer containing 20 mM HEPES (pH 7.5), 100 mM NaCl, 10 mM EDTA, 5 mM EDTA, and 5mM DTT (dithiothreitol) [28]. Relevant fractions were analyzed by sodium dodecyl sulfate–polyacrylamide gel electrophoresis and concentration was adjusted to 0.5 mg/ml and 2.4× additional FLASH was added to stabilize complex. Samples were spun 13 000 × *g* for 2 min to remove potential aggregates. Samples were kept on ice prior to grid preparation. The Lsm11 mutant U7 snRNP with H2a* was prepared following the same protocol.

### EM sample preparation and data collection

UltraAuFoil 300 mesh 1.2/1.3 gold grids (Quantifoil) were glow-discharged for 14 s with Argon and O_2_ prior to sample application. A 3.5 μl aliquot of the sample at 0.5 mg/ml concentration was applied to the glow-discharged grids. After 5 s, grids were blotted for 2 s with 55/20 mm filter paper (TED PELLA) at a blot force of −2 and plunged into cooled liquid ethane with a Vitrobot Mark IV (Thermo Fisher Scientific) set to 22°C with 100% humidity. The quality of the grids was screened using a Glacios microscope at the Columbia University Cryo-electron Microscopy Center.

A total of 8383 image stacks of the U7 machinery containing mutant Lsm11 were collected on two Titan Krios electron microscopes (Krios 1 and Krios 3) in the Simons Electron Microscopy Center at the New York Structural Biology Center using Leginon [30]. Movies were recorded with a Gatan K3 direct electron detector at a magnification of 105 000 with a pixel size of 0.844 Å and defocus from −1 to −2 μm. Exposure of 1.4 s was dose-fractionated into 40 frames (35 ms per frame), resulting in a total exposure of 56.10 e^–^/Å^2^.

A total of 6148 movies of the U7 machinery containing wild-type Lsm11 and methylated H2a*m(-4) RNA were collected on the Titan Krios electron microscope (Krios 2) at the Columbia University Cryo-electron Microscopy Center using Leginon. Movies were recorded with a Gatan K3 direct electron detector at a magnification of 105 000 with a pixel size of 0.825 Å and defocus from −1 to −1.5 μm. Exposure of 2.5 s was dose-fractionated into 50 frames (50 ms per frame), resulting in a total exposure of 58.1 e^–^/Å^2^.

### Cryo-EM data processing

Image stacks were motion-corrected, dose-weighted, and patch CTF parameters were determined in CryoSPARC [31]. For the mutant Lsm11 dataset, volumes generated from the Glacios screening data were used as templates for particle picking (Supplementary Fig. S1). Particles from the U7 machinery with mutant Lsm11 (2 941 335 particles) were extracted with a 400 pixel box, binned to 100 pixels, and classified by heterogeneous refinement using volumes representing the full U7 snRNP, U7 Sm ring with symplekin NTD, core U7 snRNP, HCC, and junk. After three rounds of heterogeneous refinement, particles corresponding to the full U7 machinery were re-extracted with 400 pixel box and binned to 320 pixels. Particles were subjected to two more rounds of heterogeneous refinement. To maximize the number of particles and views of the U7 machinery, blob picking was performed in parallel, using a diameter of 100–200 Å, and the particles were processed in a similar fashion. Particles from both approaches were combined for 2D classification and duplicate particles were discarded. These particles were used for nonuniform refinement using the volume corresponding to the intact U7 machinery. The resulting 3.85 Å map was used for model building. Particles lacking CPSF73 density were identified from heterogeneous refinement after template picking, giving rise to a 4.19 Å resolution map after nonuniform refinement. Combining with particles from blob picking did not lead to any improvement in this map.

For the dataset of wild-type U7 machinery in complex with methylated H2a*m(-4) RNA, template picking using volumes from Glacios screening data identified 2 167 021 particles (Supplementary Fig. S2). Particles were extracted with a 384 pixel box size with Fourier cropping to 96 pixels. Good particles were identified by 2D classifications, and new 3D volumes generated by *ab initio* reconstruction from these good particles. All the particles were then classified by heterogeneous refinement against these volumes. Particles corresponding to the full U7 machinery were used for nonuniform refinement, followed by reference motion correction, and a final nonuniform refinement, resulting in a 2.82 Å map. A mask was created from this volume to locally refine the symplekin CTD region of the map. The mask was used for particle subtraction and the resulting particles used for local refinement. This yielded a 3.01 Å locally refined map. The resulting maps were used for model building individually.

The symplekin NTD bound to U7 Sm ring was first identified in our initial cryo-EM study on the U7 snRNP (Supplementary Fig. S3). Blob picking for particles between 100 and 200 Å diameter in 9871 micrographs found 8 741 039 particles. These particles were extracted using 360 pixel box size and binned to 90 by Fourier cropping. Two-dimensional classification was used to remove junk and good 2D classes were selected for Topaz training and extraction [32]. The particles were extracted with 300 pixel box size and 100 pixel cropping. Two-dimensional classification was repeated and promising 2D classes selected for *ab-initio* reconstruction. After rounds of heterogeneous refinement, a nonuniform refinement using 236 295 particles produced a map at 3.18 Å resolution.

### Model building and refinement

Model building was started by using entry 6V4X [16] from the Protein Data Bank and the “fit in map” tool in ChimeraX [33]. Coordinates were saved and refined using phenix.real_space_refine global minimization (default), morphing and simulated annealing rama potential [34]. The models were then trimmed and adjusted to fit the EM density in Coot [35], adhering to torsion, planar peptide, trans peptide, and Ramachandran restraints. The composite map was generated in PHENIX. The statistics from the structure determination are summarized in Table 1.

**Table 1.** Cryo-EM data collection, structure refinement, and validation statistics

	U7 snRNP with Lsm11 mutant	U7 snRNP with noncleavable RNA	U7 Sm ring with symplekin NTD
**Data collection and processing**			
Magnification			
Voltage (kV)	300	300	300
Electron exposure (e^–^/Å^2^)	56.1	58.0	51.79
Defocus range (μm)	–1 to –2	–1 to –1.5	–1 to –2.5
Pixel size (Å)	0.844	0.825	1.083
Symmetry imposed	C1	C1	C1
Image stacks (no.)	8383	6148	12 554
Initial particles images (no.)	2 895 074	2 167 021	8 741 039
Final particle images (no.)	268 599	168 875	236 295
Map resolution (Å)	3.85	3.01	3.18
FSC threshold	0.143	0.143	0.143
Map sharpening B-factor (Å^2^)	−122.9	−90.6	−107.1
**Structure refinement**			
Number of protein residues	1 922	2 297	897
Number of metal ions	2	2	0
Number of atoms	16 565	19 390	7921
R.m.s. deviations			
Bond lengths (Å)	0.005	0.005	0.005
Bond angles (°)	1.2	1.1	1.2
PDB validation			
Clash score	6.8	6.43	4.16
Poor rotamers (%)	0.23	0.10	0.00
Ramachandran plot			
Favored (%)	93.91	95.89	96.51
Allowed (%)	5.93	4.06	3.49
Disallowed (%)	0.16	0.04	0.00
PDB entry code	9NB1	9NH6	9N96

## Results and discussion

### Mutations in Lsm11 segment reduce U7 snRNP cleavage activity

**Figure 2.**
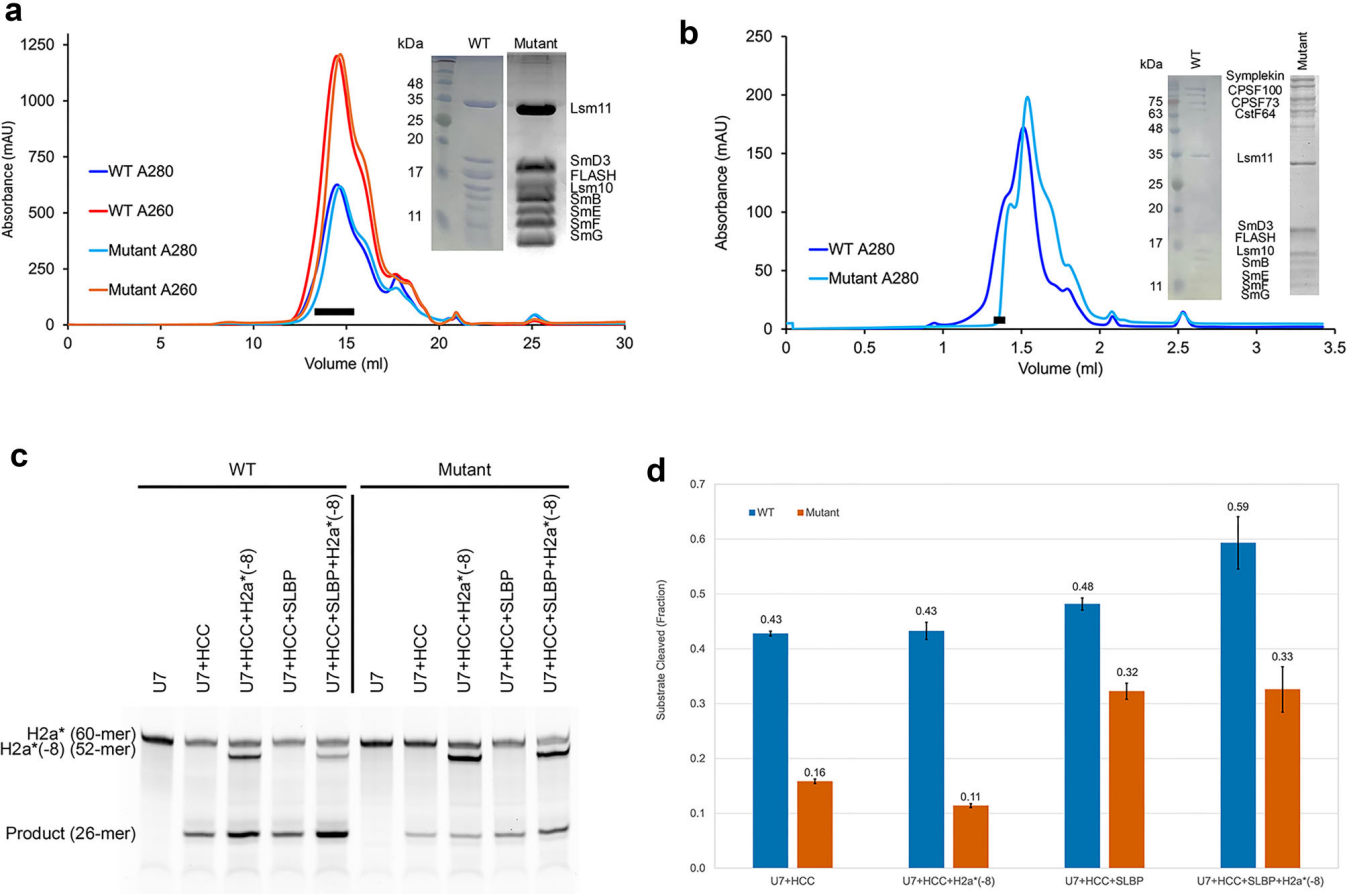
The Lsm11 segment is important for the cleavage activity of U7 snRNP. (**a**) Gel filtration profiles for the assembly of the wild-type and mutant U7 Sm ring. The black bar indicates fractions that were pooled for the U7 Sm ring. Inset: SDS gel of the samples. (**b**) Gel filtration profiles for the assembly of the entire wild-type and mutant U7 snRNP. The second, larger peak is for excess U7 Sm ring in the mixture. (**c**) Cleavage assays with the wild-type (WT) and Lsm11 mutant U7 snRNP using model substrates H2a* and H2a*(-8). (**d**) Fraction of substrate RNA cleaved for wild-type and mutant U7 snRNP. The reactions were carried out in triplicates, and error bars are standard deviations.

We introduced six mutations in the Lsm11 segment to disrupt its interactions with CPSF73, R109E/I110A/R112E/L113A/R114E/L116A (Fig. 1e). Positively charged residues (Arg) were replaced with similar-sized negatively charged residues (Glu) and hydrophobic amino acids were mutated to alanine to reduce hydrophobic interactions. We reconstituted the machinery following the protocols described earlier, assembling the U7 Sm ring first (with wild-type or the sextuple mutant Lsm11) and then the entire machinery [16, 28]. The mutations did not affect the assembly of the U7 Sm ring (Fig. 2a) or the entire U7 snRNP (Fig. 2b), with the mutant producing essentially identical profiles on a gel filtration column as the wild-type machinery.

We next compared the cleavage activity of the wild-type and mutant machineries toward model histone pre-mRNA substrates. The machineries were reconstituted with a 60-mer H2a* model substrate, with a 5′ FAM label (Supplementary Table S1). This H2a* substrate was at 1:1 stoichiometry with the U7 snRNA. To assess whether the mutations affected the recycling of the machinery to cleave additional substrates (multiple turnover), we also added a 52-mer model substrate [H2a*(-8), with a 5′ FAM label] in some of the reactions. This H2a*(-8) substrate differed from H2a* by lacking 8 nucleotides at the 3′ end (Supplementary Table S1), beyond the HDE–U7 duplex. It ran at a different position in the gel, allowing us to monitor its cleavage, although it generated the same 5′ product as did H2a*. This substrate was added at the beginning of the reaction, but the U7 snRNP would not have access to it until the 60-mer H2a* substrate that was reconstituted with the machinery was cleaved.

Our biochemical assays showed that the wild-type machinery had good cleavage activity toward both the H2a* substrate that was reconstituted with the machinery and the H2a*(-8) substrate that was added separately (Fig. 2c and d). SLBP generally stabilizes the machinery and promotes cleavage, and we observed an activating effect on the cleavage of both H2a* and H2a*(-8). In contrast, the sextuple mutant machinery had ~3-fold lower activity toward the H2a* substrate as compared to the wild-type in the absence of SLBP, and the addition of H2a*(-8) resulted in only minor increases in product formation (Fig. 2c), suggesting that the mutant had low activity toward the H2a*(-8) substrate such that the overall substrate turnover was actually reduced (Fig. 2d). The presence of SLBP led to a two-fold enhancement of the cleavage activity toward the H2a* substrate, and an even greater activity enhancement toward the H2a*(-8) substrate. The cleavage activity of the mutant was only 30%–40% lower compared to that of the wild-type machinery. SLBP likely provided additional stabilization of the mutant machinery.

We also created an Lsm11 mutant where this segment was deleted (residues 102–120) and had very similar observations on its cleavage activity as the sextuple mutant (Supplementary Fig. S4).

The weak activity of the mutant machinery, especially toward additional substrates, suggested a possibility that it might not degrade the downstream cleavage product (DCP) after the cleavage reaction, thereby blocking the incorporation of the next pre-mRNA substrate. Our previous studies have shown that CPSF73 functions as a 5′-3′ exonuclease to degrade the DCP after the cleavage reaction [28, 36]. We tested the activity of the wild-type and Lsm11 sextuple mutant U7 snRNP assembled with a doubly labeled H2a*(-8) substrate (5′ TAMRA and 3′ FAM), and found that the mutant was able to degrade the DCP (Supplementary Fig. S4). Therefore, the low activity of the mutant U7 snRNP toward additional substrates was due to a defect in substrate loading and/or cleavage, rather than blockage of the CPSF73 active site by undegraded DCP.

Overall, our studies demonstrate that the Lsm11 segment that contacts CPSF73 is important for the cleavage activity of the U7 snRNP. This segment is connected to the rest of Lsm11 through long, flexible linkers, allowing it to function independently of the rest of the protein. The N-terminal end of Lsm11 interacts with FLASH, and the CTD is incorporated into the Sm ring (Fig. 1a). Whether this segment that contacts CPSF73 has other functions in the U7 snRNP will require further studies.

### The Lsm11 segment helps to stabilize CPSF73 in U7 snRNP

**Figure 3.**
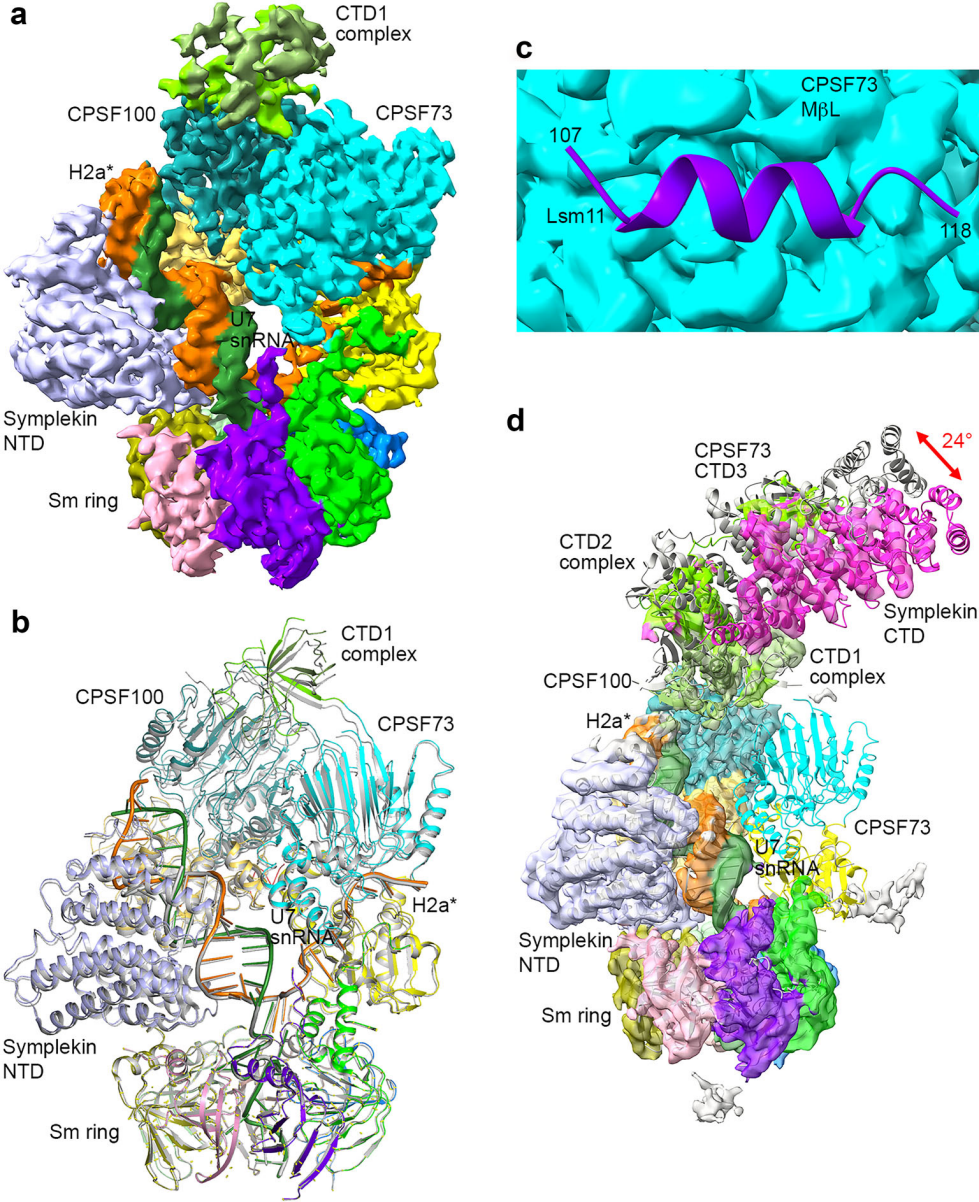
Structures of U7 snRNP with six mutations in the Lsm11 N-terminal segment. (**a**) Cryo-EM density at 3.7 Å resolution of the core of the mutant U7 snRNP, colored by subunits. EM density for the H2a* RNA substrate (orange) is visible in the active site of CPSF73. The U7 snRNA density is colored dark green. (**b**) Overlay of the structure of the mutant U7 snRNP core (in color) with that of the wild-type U7 snRNP core (in gray). The overlay is based on the U7 Sm ring. (**c**) Cryo-EM density (cyan surface) of the mutant U7 snRNP core in the expected binding region of the Lsm11 N-terminal segment (purple cartoon). This segment has no EM density. (**d**) Cryo-EM density at 4.2 Å resolution (transparent surface) for another class of mutant U7 snRNP particles that lack density for the catalytic segment of CPSF73. A conformational change for the symplekin CTD is also observed compared to the earlier structure of the U7 snRNP (in gray).

We next carried out cryo-EM studies to assess the effect of the mutations in the Lsm11 segment on the structure of the U7 snRNP. We obtained an EM reconstruction at 3.8 Å resolution for the core of the U7 snRNP (Table 1 and Supplementary Fig. S1), with good density for the pre-mRNA in the CPSF73 active site. There was no density for the periphery of the machinery (Fig. 3a). We built an atomic model for this core based on the structure we reported earlier [16] (Fig. 3b and Table 1). Compared to the earlier structure, there were only small differences in the relative positions of the subunits (Fig. 3b), and the binding mode of the RNA in the active site of CPSF73 was highly similar (Supplementary Fig. S5). On the other hand, there was no density for the Lsm11 segment in this EM map (Fig. 3c), confirming that the mutations had disrupted the interaction with CPSF73. These observations indicated that the Lsm11 segment was not essential for U7 snRNP to adopt the active conformation, consistent with our assay results. No EM density was observed for FLASH and SLBP, and the mechanism how SLBP stimulated the activity of this mutant (Fig. 2c and d) will require further studies.

During the EM data processing, we identified another class of particles and obtained an EM reconstruction at 4.2 Å resolution (Supplementary Fig. S1). This map lacked any visible density for the catalytic segment of CPSF73 (MβL and β-CASP domains), while the rest of the U7 snRNP core (including CTD1 of CPSF73, which was in complex with CTD1 of CPSF100) had EM density (Fig. 3d). In fact, some density was also observed for the CTD2 complex of CPSF73–CPSF100, CTD3 of CPSF73, and the N-terminal part of the symplekin CTD, and models of these domains could be built into the EM density based on those from AlphaFold [17]. The overall structures of the CTD1 and CTD2 complexes of CPSF73–CPSF100 were similar to those from AlphaFold, with RMS (root mean square) distance of ~0.5 Å for equivalent Cα atoms. The relative positions between the CTD1 and CTD2 complexes were however somewhat different compared to the AlphaFold model. The EM density for CTD3 was weaker, and only its two helices could be built in with confidence. Compared to the U7 snRNP structure we reported earlier, the symplekin CTD and the associated CTD2 complex of CPSF73–CPSF100 and CTD3 of CPSF73 had a different orientation and position, equivalent to a 24° rotation (Fig. 3d), illustrating the flexibility in this peripheral region of the machinery.

This structure could represent a failure to correctly assemble the U7 snRNP and activate CPSF73 to the open conformation. This class of particles appeared to be more abundant than the class that showed CPSF73 density, ~70 000 particles for this class compared to ~46 000 particles for the class with CPSF73 (Supplementary Fig. S1). As a comparison, we did not observe particles lacking CPSF73 density in our earlier structure of the wild-type, active machinery [16]. The observations with these two classes of particles suggest that a function of the Lsm11 segment is to stabilize the position of CPSF73 in the U7 snRNP, helping to keep it in place for RNA binding and catalysis. The absence of this Lsm11 segment makes it more likely for the CPSF73 catalytic segment to be flexible, and such a configuration of the machinery would not be able to efficiently cleave the pre-mRNA substrate, explaining the molecular basis for our biochemical observations.

### CPSF73 in an open conformation without RNA in the active site

**Figure 4.**
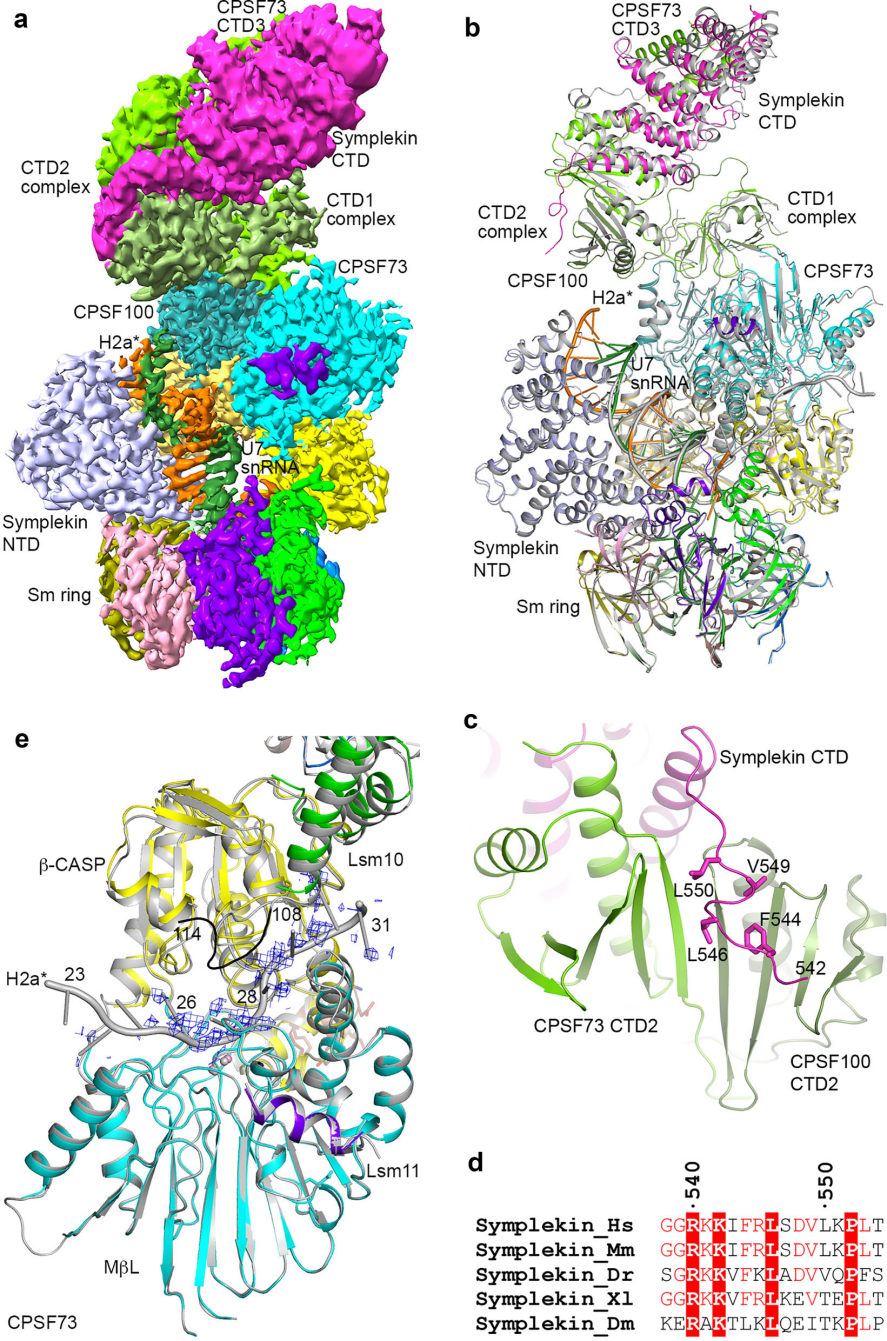
CPSF73 in an open state but without RNA bound in the active site. (**a**) Composite EM map of the core of the U7 snRNP in complex with H2a*m(-4) pre-mRNA and a part of the periphery after local refinement. The density for symplekin CTD is not as sharp due to the lower resolution. (**b**) Overlay of the structure of U7 snRNP in complex with H2a*m(-4) (in color) with that in complex with H2a* reported earlier (in gray). (**c**) An extended segment at the N-terminal end of the symplekin CTD is located over the β-sheet of the CPSF73–CPSF100 CTD2 complex. Several hydrophobic residues interact with the CPSF100 CTD2 β-sheet. (**d**) Sequence conservation of this extended segment just before the symplekin CTD. Dm: fruit fly. (**e**) EM density for the active site region of CPSF73 in the complex with H2a*m(-4). An overlay of CPSF73 in this structure with that in complex with H2a* (in gray) is also shown. There is no EM density for the H2a*m(-4) RNA in the active site.

We observed earlier that methylation of the 2′ hydroxyl of the ribose of five nucleotides spanning the cleavage site of the histone pre-mRNA abolished the cleavage by the machinery, and such an RNA was used to identify all components of the U7 machinery [8, 11, 21]. However, how this methylation blocks the cleavage is not known, as the 2′ hydroxyl does not participate in the hydrolysis reaction [16]. We reconstituted a wild-type U7 snRNP bound to this methylated, noncleavable H2a* pre-mRNA, H2a*m(-4) (lacking 4 nucleotides at the 3′ end compared to H2a*; Supplementary Table S1) and obtained a cryo-EM density map at 2.8 Å resolution for the core of this machinery (Table 1 and Supplementary Figs S2 and  S6a). The EM analysis also showed weak density for the N-terminal segment of the symplekin CTD and associated segments from CPSF73 and CPSF100, and the density was improved through a local refinement in this region. This allowed us to generate a composite EM map that covered a larger portion of the U7 snRNP (Fig. 4a).

The overall structure of this U7 snRNP was similar to that we reported earlier (Fig. 4b). A longer duplex was built based on the EM map (as was the case with the Lsm11 mutant; Fig. 3b), although the EM density at the end of the duplex (3′ end of the H2a* and 5′ end of the U7 snRNA) became weaker. An extended segment at the N-terminal end of the symplekin CTD was placed over the β-sheet of the CPSF73–CPSF100 CTD2 complex (Fig. 4a). Residues Phe544, Leu546, Val549, and Leu550 primarily interacted with the CPSF100 CTD2 β-sheet (Fig. 4c). These residues are highly conserved among symplekin orthologs (Fig. 4d).

However, one major difference was that there was no EM density for the H2a* RNA in the CPSF73 active site, even though it was also in an open conformation (Fig. 4e). In addition, no EM density was observed for the C-terminal end of Lsm10 (residues 108–114) consistent with its interaction with the RNA. Therefore, the methylation of the ribose 2′ hydroxyl groups may have prevented the binding of the substrate into the CPSF73 active site, which would explain why methylation abolished the cleavage of the substrate. An examination of the RNA binding mode in the CPSF73 active site [16] indicated that methylation of the 2′ hydroxyl of the two nucleotides just 3′ to the cleavage site could introduce steric clashes with CPSF73.

Interestingly, the EM analysis also revealed a class of particles that lacked density for the catalytic segment of CPSF73 (Supplementary Fig. S6b), similar to what we observed for the Lsm11 mutant (Fig. 3d). The symplekin CTD had a smaller movement compared to our earlier structure [16], only 10° rotation. In addition, we were only able to identify ~38 000 particles for this class, compared to ~169 000 particles for the class that contained CPSF73 density, suggesting that the majority of the U7 snRNP had ordered CPSF73 catalytic segment.

### A helix at the C terminus of CstF77 is bound to HCC

**Figure 5.**
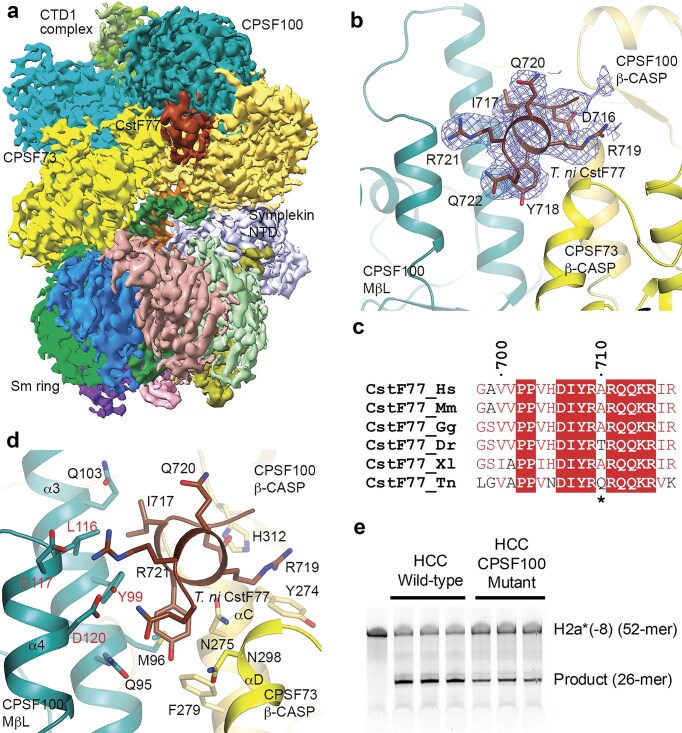
The C terminus of CstF77 is present in the U7 snRNP. (**a**) EM density at 2.8 Å resolution of the U7 snRNP core in complex with methylated, noncleavable H2a*m(-4) RNA. The density for the C terminus of CstF77 is in chocolate color. (**b**) EM density and detailed interactions between the C terminus of *T. ni* CstF77 and human CPSF73–CPSF100. (**c**) Sequence conservation of residues at the C terminus of CstF77. Residue 710 of human CstF77 highlighted with the asterisk. Tn: *Trichoplusia ni* (insect). (**d**) Detailed interactions between the *T. ni* CstF77 C-terminal segment and CPSF73–CPSF100. The four CPSF100 residues mutated in the quadruple mutant are labeled in red. (**e**) Cleavage assays with wild-type U7 snRNP and CPSF100 quadruple mutant U7 snRNP (in triplicate). The mutant has ~30% lower cleavage activity than the wild-type U7 snRNP.

In the U7 snRNP structure that we reported earlier [16], there was a small piece of EM density at the interface between CPSF73 and CPSF100 of HCC that we could not explain. The density had the feature of a short helix, but the apparent amino acid sequence of this segment did not seem to match any of the proteins that were expected to be in the sample, although this segment did not have a sufficient number of residues to allow a confident sequence comparison. This density was also observed in the new EM maps reported here, for the Lsm11 mutant U7 snRNP and the wild-type machinery with noncleavable pre-mRNA (Fig. 5a). Aided by AlphaFold-Multimer [22, 23], we found that this density corresponded to residues 715–722 near the C-terminus of CstF77 (Fig. 5b). Specifically, this was *T. ni* CstF77 (UniProt entry A0A7E5WIN8, also annotated as protein suppressor of forked) that copurified with HCC from insect cells, as we did not express human CstF77 in these experiments. The sequences of *T. ni*, human, and other CstF77 homologs are highly conserved in this region (Fig. 5c). However, Ala710 of human CstF77 is replaced by Gln720 in *T. ni* CstF77, and there was clear density for this larger side chain in the EM map (Fig. 5b), giving strong evidence that this segment was truly from *T. ni* CstF77.

The total buried surface area was 570 Å^2^ for the CstF77 segment in the complex. Residues Ile717 and Tyr718 (Ile707 and Tyr708 in human CstF77) were in the center of the interface with CPSF100 (Fig. 5d), with buried surface area of 120 and 140 Å^2^, respectively. On the other hand, *T. ni* Gln720 had no contribution to the buried surface area in the interface, suggesting that the C terminus of human CstF77 would also be able to interact with CPSF73–CPSF100 in a similar fashion.

The CstF77 binding site was on the opposite face of CPSF73–CPSF100 relative to the active site of CPSF73 (Supplementary Fig. S7), at a distance of ~40 Å, and therefore this segment of CstF77 was not expected to directly contribute to the cleavage activity of CPSF73. The CstF77 residues primarily interacted with the MβL and β-CASP domains of CPSF100, but the segment was also located next to the β-CASP domain of CPSF73 (Fig. 5d). In the EM reconstruction that had no density for the catalytic segment of CPSF73 (Fig. 3d), EM density for this CstF77 segment was still observed, suggesting that interactions with CPSF100 were sufficient for the binding of this segment.

To test the importance of this CstF77 binding site in HCC, we generated the Y99A/L116A/D117A/D120A quadruple mutant of CPSF100. These four residues were on the surface of CPSF100 and had the most contacts with CstF77 (Fig. 5d). They did not contact CPSF73, and their mutation was not expected to affect the structure of CPSF100 or interfere with the CPSF100–CPSF73 interface. We purified the mutant HCC following the same protocol as that for the wild-type HCC, and tested the cleavage activity of this mutant HCC against the H2a*(-8) substrate. We observed an ~30% decrease in the activity of this mutant (Fig. 5e), suggesting that this CstF77 binding had a small effect on the cleavage activity of HCC.

This CstF77 segment survived three column purification steps during the EM sample preparation: nickel agarose and anion exchange for HCC purification and gel filtration for the entire U7 snRNP. The EM density for this segment was at a level comparable to residues in CPSF73 and CPSF100 (Fig. 5a), suggesting that this segment was at full (or nearly full) occupancy in the particles that were used to produce the EM map. On the other hand, SDS gels of our purified HCC sample did not show a band with an intensity comparable to the proteins in HCC at the expected molecular weight for full-length *T. ni* CstF77 (84 kDa) (Supplementary Fig. S8a). A possible explanation for this was that full-length CstF77 was present at sub-stoichiometric levels in the purified protein, but most of the complexes that contributed to the final EM map contained CstF77.

We expressed and purified the C-terminal end of human CstF77 (residues 666–717) as an MBP fusion protein and carried out amylose resin pull-down assays. This segment covers the entire C-terminal end of CstF77 beyond the region that interacts with CstF64. The MBP–CstF77 fusion protein was not able to pull down more purified HCC compared to the negative control of amylose beads alone (Supplementary Fig. S8b). Separately, we expressed and purified the same C-terminal segment of human CstF77 as a SUMO fusion protein and added this sample into the cleavage reaction. We did not observe a significant effect on the cleavage activity, even with CstF77 at 10-fold molar ratio (Supplementary Fig. S8c). We also co-expressed this C-terminal segment of human or *T. ni* CstF77 as an MBP fusion protein together with HCC in insect cells but did not observe significant amount of MBP–CstF77 in the purified HCC sample, even though MBP–CstF was expressed at good levels (Supplementary Fig. S8d).

Overall, our studies indicated that the C-terminal segment of CstF77 could bind to CPSF100 in HCC, which had a small effect on the cleavage activity of the U7 snRNP. The C-terminal segment alone did not appear sufficient for binding to HCC, as it could not pull down HCC that was purified or co-expressed. It might be possible that additional region(s) of CstF77 were also involved in the interaction with HCC, but these regions of contact were flexible in structure and hence were not observed in the EM density. CstF64 is a subunit of HCC through its interaction with symplekin, but the segment of CstF64 that interacts with CstF77 is also required for CstF64 to interact with symplekin. Therefore, a different region of CstF77 is likely involved in the interaction with HCC.

### A structure of U7 Sm ring with symplekin NTD bound to the HDE–U7 duplex

**Figure 6.**
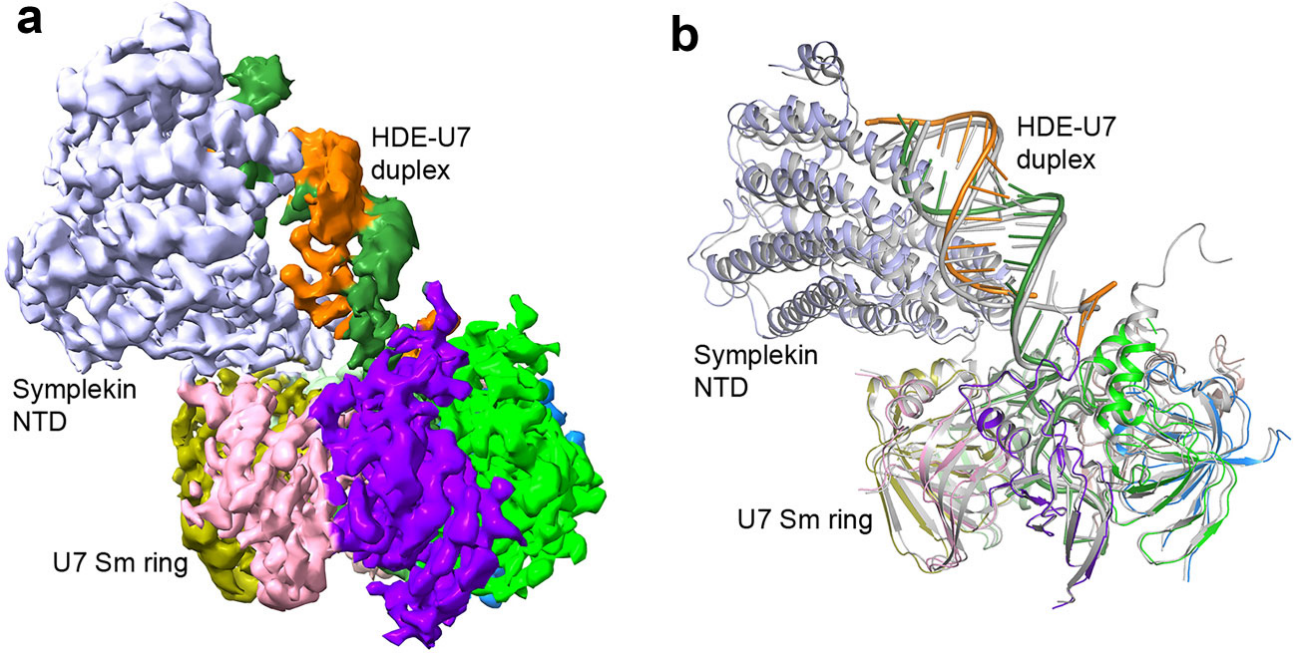
A structure of U7 Sm ring with the symplekin NTD and the HDE–U7 duplex. (**a**) EM density at 3.2 Å resolution of the U7 Sm ring with the symplekin NTD and the HDE–U7 duplex. Three nucleotides of the pre-mRNA (orange) bound to the Sm ring is also ordered. (**b**) Overlay of the structure of U7 Sm ring with the symplekin NTD and the HDE–U7 duplex (in color) with the equivalent region of the U7 snRNP (in gray). The superposition is based on the Sm ring.

In our EM analysis of both the Lsm11 mutant and the noncleavable RNA datasets, we observed another class of particles that were much smaller than the U7 snRNP, which appeared to correspond to the Sm ring (Supplementary Fig. S3). We obtained an EM map at 3.2 Å resolution using the particles from a different Lsm11 mutant dataset (Fig. 6a and Table 1). The final reconstruction contained the Sm ring, the HDE–U7 duplex, the Sm motif of the U7 snRNA, and three additional nucleotides of the pre-mRNA bound to the Sm ring, and the symplekin NTD (Fig. 6b), but the rest of the protein factors and RNA were flexible. The location of the symplekin NTD, the conformation of the HDE–U7 duplex, and its interaction with the symplekin NTD were similar to those in the U7 snRNP that we reported earlier [16]. We do not know at the current time whether this structure represents a physiologically relevant state of the machinery or it is an artifact, for example of the vitrification process during EM grid preparation.

Our studies highlight an important role for the conserved helical segment in the N-terminal extension of Lsm11, which would provide a molecular basis for the high sequence conservation of residues in this helix. The helix helps to stabilize the catalytic segment of CPSF73, helping to keep it in place for RNA binding and cleavage. This segment could also contribute to other functions of U7 snRNP, and further studies of this U7 snRNP mutant in cells would be needed to assess this possibility.

The surface of CPSF73 MβL domain for the binding of the helical segment in the N-terminal extension of Lsm11 overlaps with that for the binding of Mpe1 by the yeast CPSF73 homolog Ysh1 [18]. In addition, this surface of the MβL domain of the CPSF73 paralog INTS11 mediates the interaction with INTS13 in Integrator (Supplementary Fig. S9) [37]. This is a part of the interface between the tail module (INTS10–INTS13–INTS14–INTS15) and the rest of Integrator, although it is not known whether INTS13 can affect the catalytic activity of INTS11. Overall, the observations on CPSF73 and INTS11 suggest that this surface of the MβL domain may have important roles in the activity of these nucleases.

Our studies have revealed a region of contact between CstF77 and HCC. The binding of CstF77 to HCC may also have implications for the equivalent mCF in canonical pre-mRNA 3′-end processing. A high-resolution structure of mCF in the canonical machinery has not been obtained yet, therefore it has not been established whether the C-terminal end of CstF77 is associated with CPSF73–CPSF100 in this factor. Given our observations on the HCC, it is highly likely that this CstF77 segment is also associated with mCF and it may also have an effect on the canonical machinery.

Our observations with the noncleavable pre-mRNA demonstrate that CPSF73 can achieve an open conformation in the U7 snRNP even without any RNA being bound in its active site, consistent with the idea that the activation of CPSF73 is facilitated by the other protein factor(s) (especially Lsm10), independent of RNA binding to its active site, which prepares it to accommodate the RNA substrate for cleavage. Overall, our studies reported here have revealed additional mechanisms for modulating the activity of the U7 snRNP—the Lsm11 N-terminal helical segment helps to hold CPSF73 in the correct position for catalysis; the activation of CPSF73 is facilitated by other protein factor(s) independent of substrate RNA binding to its active site; CstF77 C-terminal segment is associated with HCC and has a small effect on the U7 snRNP cleavage activity and possibly also affect the canonical machinery.

## Supplementary Material

gkaf1442_Supplemental_File

## Data Availability

The atomic coordinates and the EM maps have been deposited at the Protein Data Bank, with accession codes 9NB1 (Lsm11 mutant), 9NH6 (noncleavable RNA), and 9N96 (U7 Sm ring with symplekin NTD).
