# Supplementary material for: An N-terminal helix of Lsm11 stabilizes CPSF73 in U7 snRNP for histone pre-mRNA 3′-end processing
Source: Nucleic Acids Res. 2026 Jan 6;54(1):gkaf1442. doi: 10.1093/nar/gkaf1442 (PMC12774660; doi:10.1093/nar/gkaf1442)
Supplement: gkaf1442_Supplemental_File [file gkaf1442_supplemental_file.pdf]

Supplementary Table 1

Sequences of DNA and RNA oligos used in this study

**DNA**	**Sequence**
CstF77 F1	TATGCTAGCTGTAGAAGGCAACGGCCCCGTGGAAAGTAATGC<br>AGTACTCACCAAGGCCGTCAAAAGGCCCAACGAGGATTCAGA
CstF77 R1	TCCTCGTTGGGCCTTTTGACGGCCTTGGTGAGTACTGCATTA<br>CTTTCCACGGGGCCGTTGCCTTCTACAGCTAGCA
CstF77 F2	TGAAGATGAAGAAAAGGGAGCCGTTGTCCCCCCTGTTCATGA<br>CATTTACAGAGCACGGCAGCAGAAGCGGATTCGGTAAC
CstF77 R2	TCGAGTTACCGAATCCGCTTCTGCTGCCGTGCTCTGTAAATGTCA<br>TGAACAGGGGGGACAACGGCTCCCTTTTCTTCATCTTCATCTGAA
**RNA**	**Sequence**
U7 snRNA<br>(1-63)	CA**GUGUUACAGCUCUUU**UAGAAUUUGUCUAGUAGGCUUU<br>CUGGCUUUUUACCGGAAAGCCCCU
H2a*<br>(60-mer)	FAM-CCAAAGGCUCUUUUCAGAGCCACCCA↓CUGAAUCAGAU<br>**AAAGAGCUGUAACAC**GGUAGCCA
H2a*(-8)<br>(52-mer)	FAM-CCAAAGGCUCUUUUCAGAGCCACCCA↓CUGAAUCAGAU<br>**AAAGAGCUGUAACAC**
H2a*m(-4)<br>(methylated<br>56-mer)	FAM-CCAAAGGCUCUUUUCAGAGCCACmCmCmA↓mCmUGAA<br>UCAGAU**AAAGAGCUGUAACAC**GGUA

Down arrows indicate site of cleavage, underlined nucleotides are in the stem-loop, bold nucleotides in H2a* sequences comprise the histone downstream element (HDE) and form 15 Watson Crick base-pairs with complementary bold nucleotides in U7 snRNA.

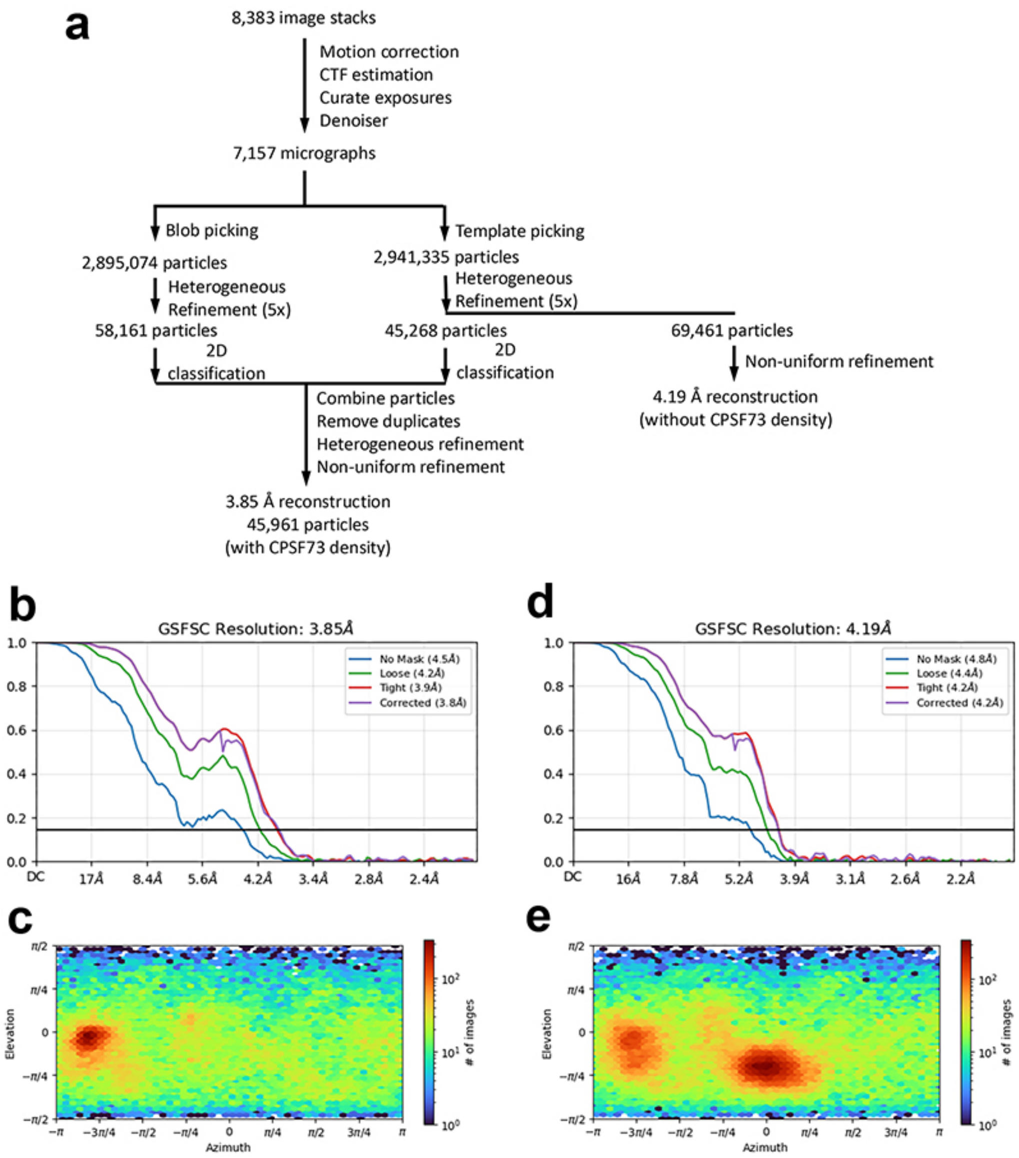

Supplementary Fig. 1. Single-particle cryo-EM analysis of the human U7 snRNP with mutant Lsm11. **(a).** Flow chart of the cryo-EM data processing. **(b).** Fourier shell correlation curves for the final overall reconstruction for particles containing CPSF73 density. **(c).** Orientations of particles used in the refinement for the final cryo-EM reconstruction. **(d).** Fourier shell correlation curves for the final overall reconstruction for particles lacking CPSF73 density. **(e).** Orientations of particles used in the refinement for the final cryo-EM reconstruction.

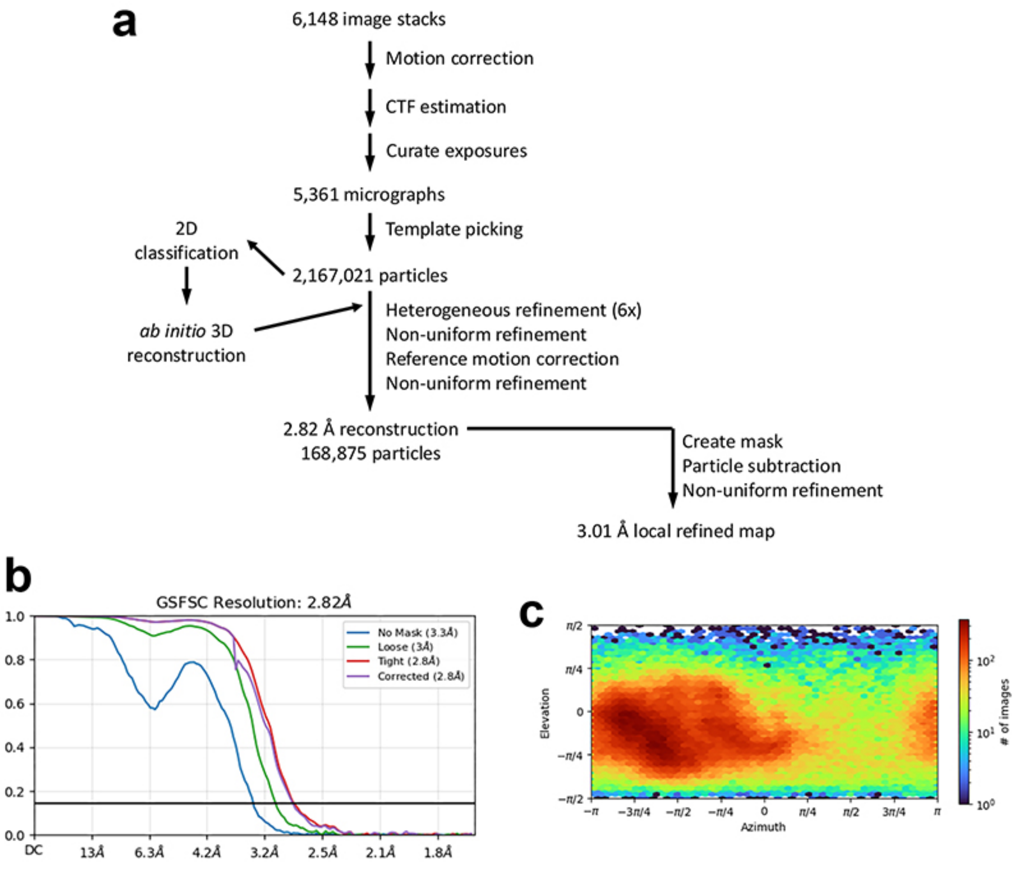

Supplementary Fig. 2. Single-particle cryo-EM analysis of U7 snRNP in complex with a methylated, noncleavable pre-mRNA. **(a).** Flow chart of the cryo-EM data processing. **(b).** Fourier shell correlation curves for the final overall reconstruction. **(c).** Orientations of particles used in the refinement for the final cryo-EM reconstruction.

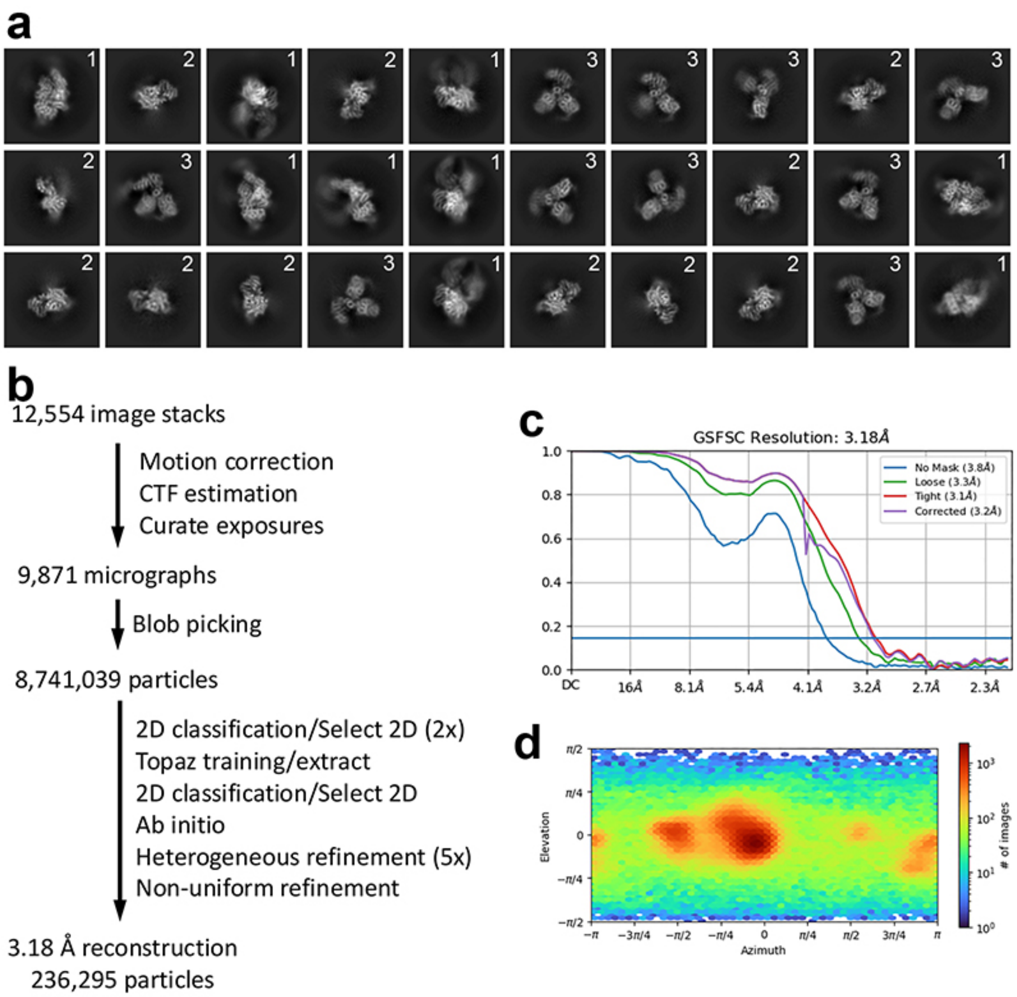

Supplementary Fig. 3. Single-particle cryo-EM analysis of the U7 Sm ring with symplekin NTD. **(a).** Select 2D class averages based on the EM analysis. Three types of particles could be recognized: 1 – U7 snRNP; 2 – U7 Sm ring with symplekin NTD; 3 – HCC trilobal structure. **(b).** Flow chart of the cryo-EM data processing. **(c).** Fourier shell correlation curves for the final overall reconstruction. **(d).** Orientations of particles used in the refinement for the final cryo-EM reconstruction.

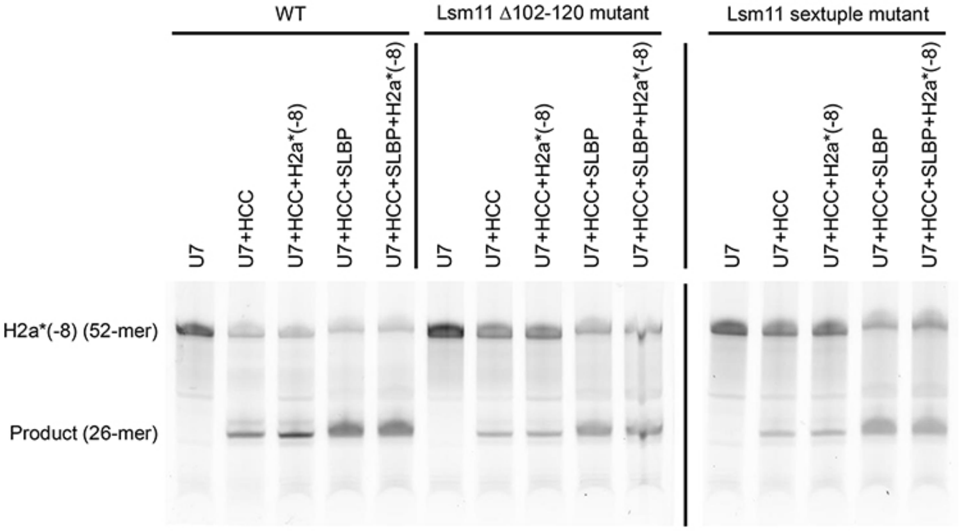

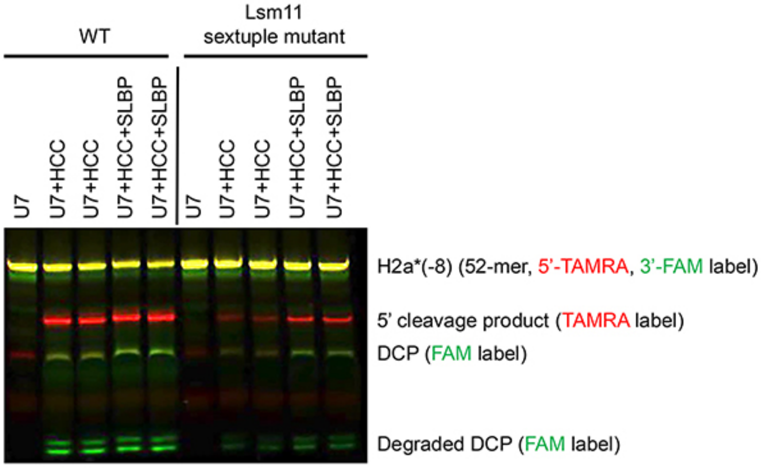

Supplementary Fig. 4. (***Top***) Cleavage assays with the wild-type (WT), Lsm11 Δ102-120 deletion mutant, and Lsm11 sextuple mutant (R109E/I110A/R112E/L113A/R114E/L116A) U7 snRNP using the model substrate H2a*(-8), which was reconstituted with the machinery. In some of the reactions (3rd and 5th lanes for each machinery), extra H2a*(-8) was added separately. The observations with the deletion mutant parallel those for the sextuple mutant. (***Bottom***) Cleavage assays with the wild-type (WT) and Lsm11 sextuple mutant U7 snRNP using the doubly labeled model substrates H2a*(-8) (5' TAMRA signal colored in red, 3' FAM signal colored in green). The mutation does not block the degradation of the downstream cleavage product (DCP). The reactions were carried out in duplicate.

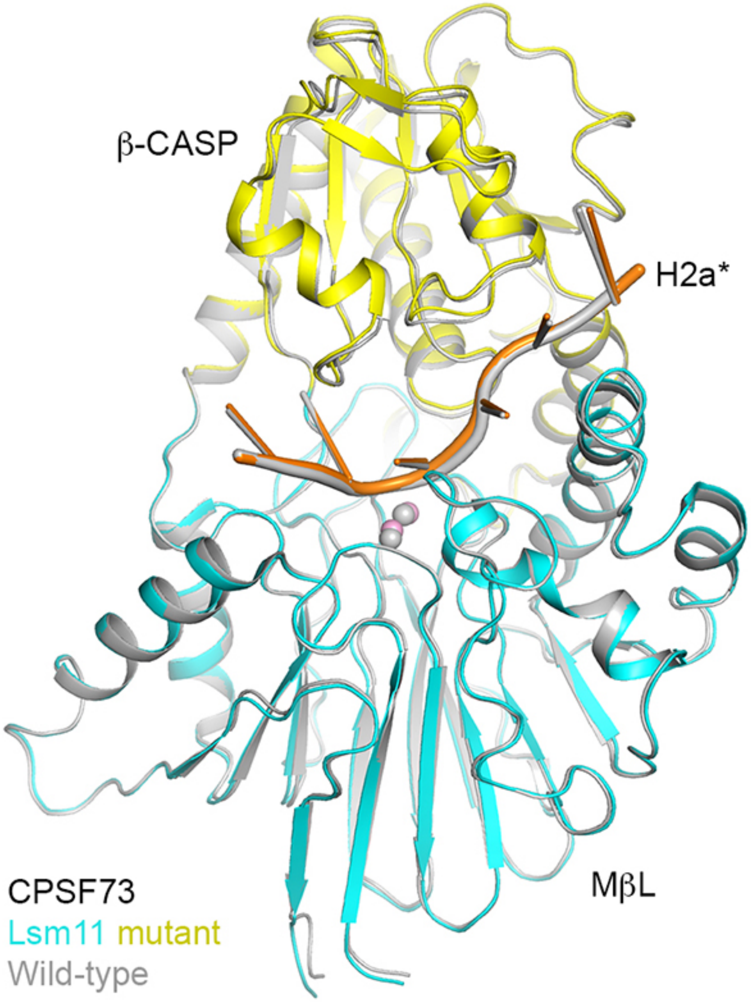

Supplementary Fig. 5. A comparison of the binding mode of pre-mRNA in the active site of CPSF73. Overlay of the binding mode of H2a* pre-mRNA in the active site of CPSF73 (in color) in Lsm11 mutant U7 snRNP with that of wild-type U7 snRNP (in gray).

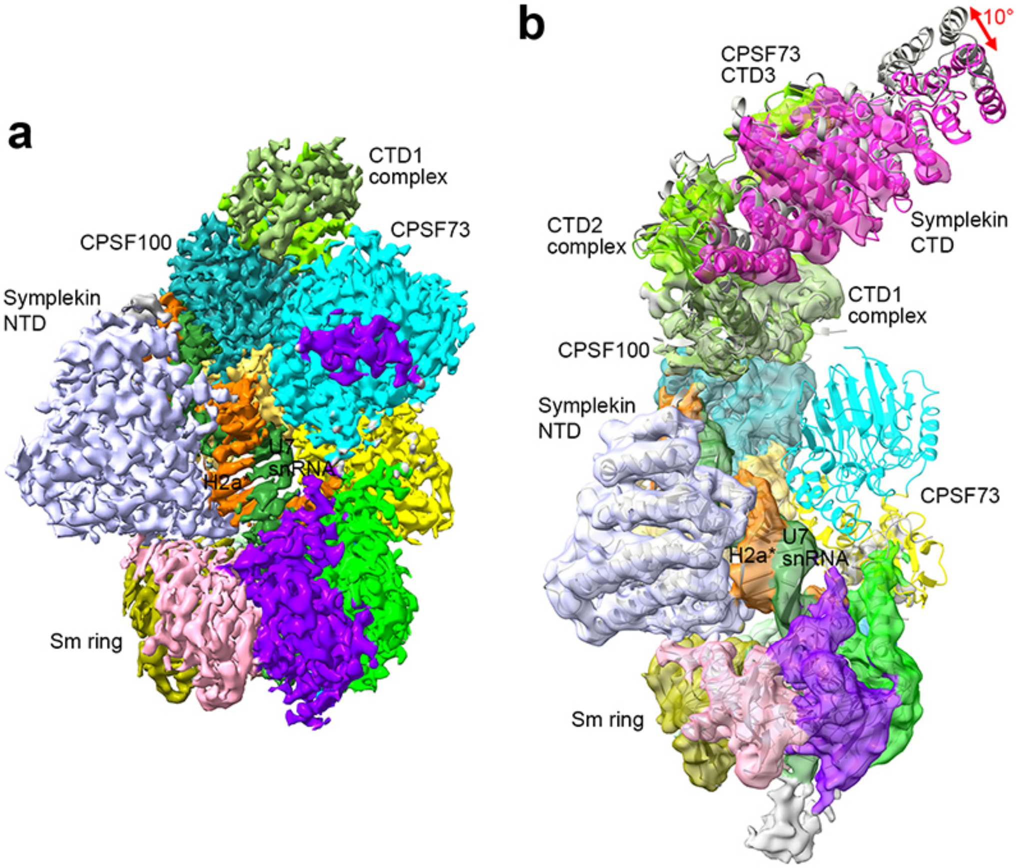

Supplementary Fig. 6. EM density of wild-type human U7 snRNP in complex with a methylated, non-hydrolyzable pre-mRNA. **(a).** EM density of the core of wild-type U7 snRNP in complex with a methylated, non-hydrolyzable H2a* pre-mRNA. The subunits are given different colors. H2a* pre-mRNA is in orange, and U7 snRNA in dark green. **(b).** EM density of the core of U7 snRNP in complex with a methylated, non-hydrolyzable H2a* pre-mRNA, for the class of particles that lack density for the CPSF73 catalytic segment. Some EM density for the symplekin CTD is observed, but with a smaller change in its orientation (10°) compared to that in the Lsm11 mutant U7 snRNP.

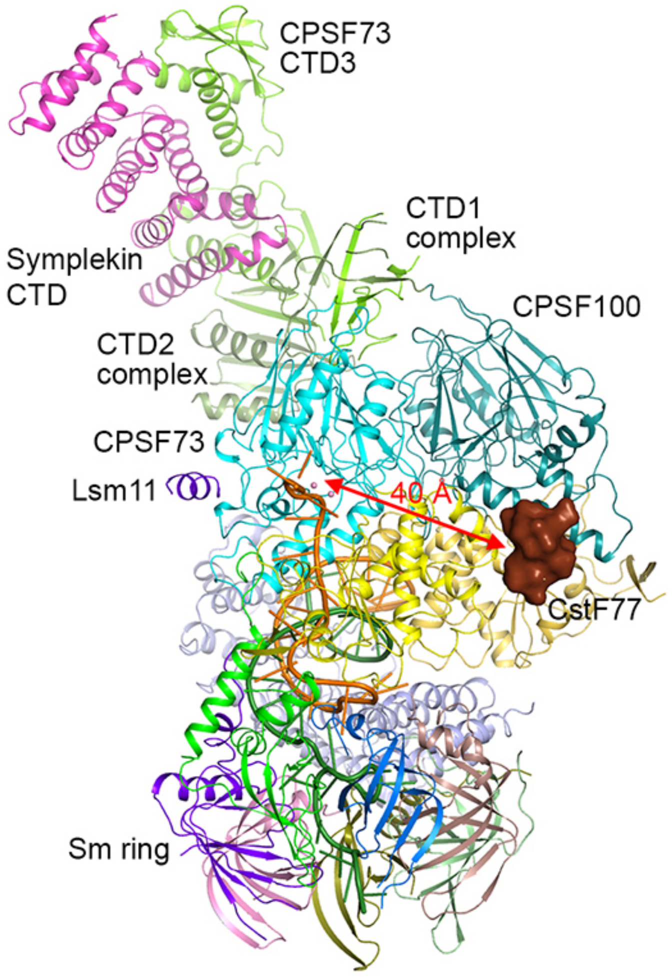

Supplementary Fig. 7. Location of the CstF77 binding site relative to the CPSF73 active site. The CstF77 peptide is shown as a surface in brown. It is located on the opposite face from the CPSF73 active site, at a distance of 40 Å (red arrow).

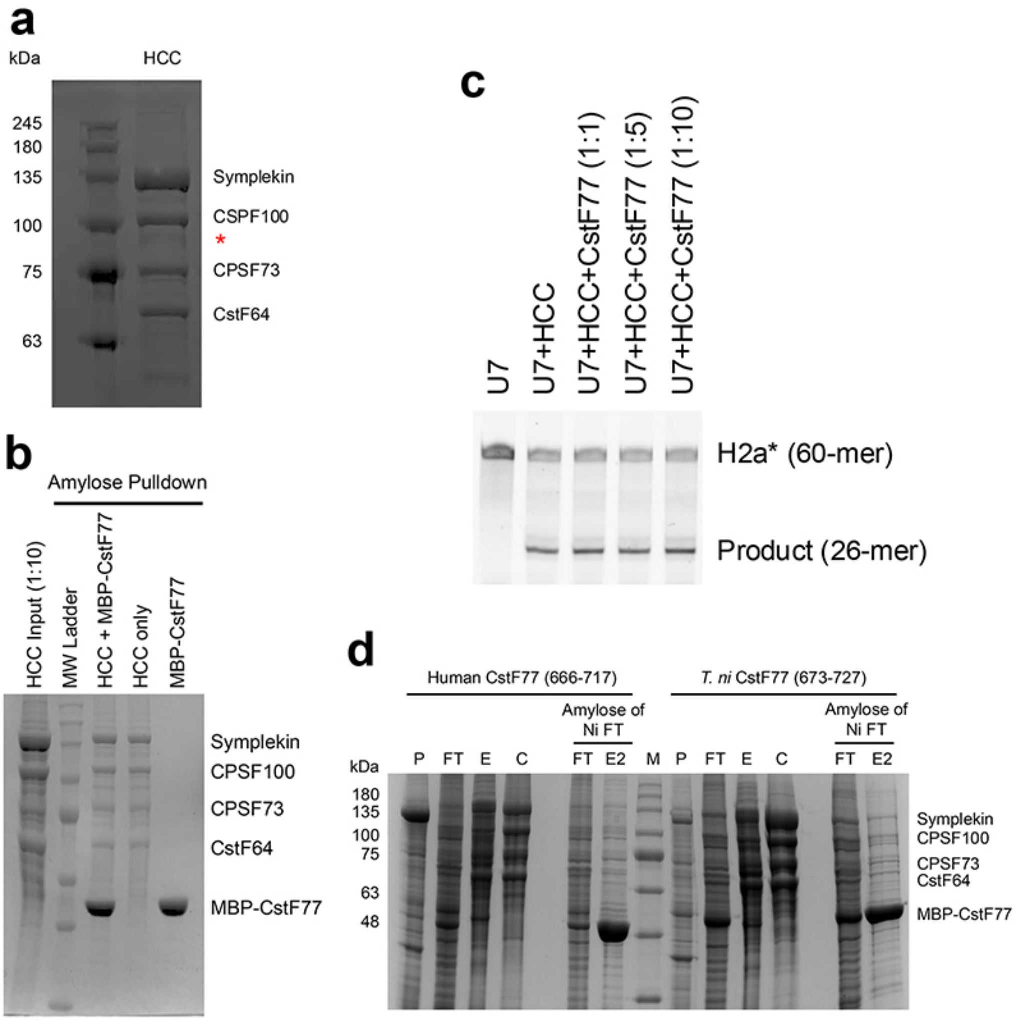

Supplementary Fig. 8. (a). SDS gel of purified HCC. Four clear bands are visible for symplekin, CPSF100, CPSF73 and CstF64. There is no band of comparable intensity at the expected molecular weight for full-length CstF77 (red asterisk). **(b)**. Amylose pulldown experiments with human MBP-CstF77 C-terminal segment immobilized on amylose beads and purified HCC. HCC shows some binding to amylose beads in the absence of MBP-CstF77. **(c)**. Cleavage assays in the presence of added human CstF77 C-terminal segment as a SUMO fusion protein at increasing molar ratios. **(d)**. Human or *T. ni* MBP-CstF77 C-terminal segment was co-expressed with human HCC in *T. ni* cells, and HCC was purified by nickel affinity chromatography. There was little indication of the presence of MBP-CstF77 in the purified HCC. The nickel flowthrough was subjected to amylose pulldown, confirming that MBP-CstF77 was expressed at high levels. P: pellet; FT: flowthrough; E: nickel elute; C: concentrated fraction after anion exchange; E2: amylose resin elute with maltose after binding the Ni elute; M: molecular weight markers. The bands are labeled on the left.

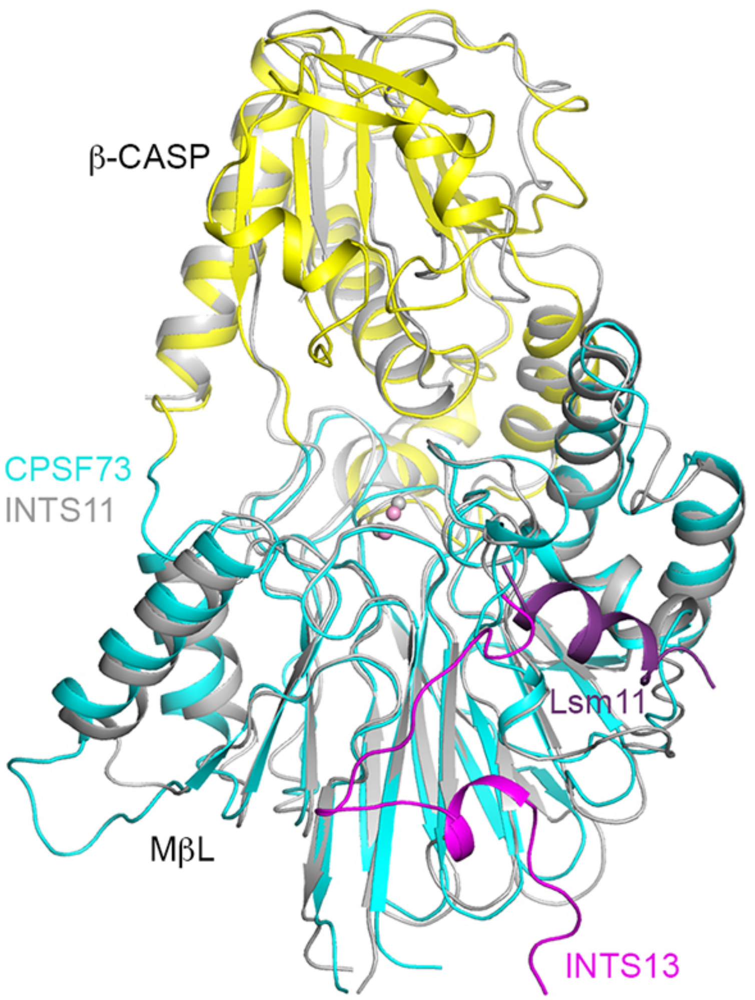

Supplementary Fig. 9. A binding site on the surface of the metallo-β-lactamase (MβL) domain of CPSF73 and INTS11. Overlay of the structures of CPSF73 (active state, cyan for the MβL domain and yellow for the β-CASP domain) in complex with the Lsm11 N-terminal segment (purple) and INTS11 (active state, gray) in complex with a segment from INTS13 (magenta). Lsm11 and INTS13 are located on the same surface of the MβL domain.
